# Complications in Diagnosis of Susceptible Cases of Fragile X Syndrome

**Published:** 2018-07

**Authors:** Reza JAFARZADEH ESFEHANI, Mahtab DASTPAK, Mohammad Reza MIRINEZHAD, Ariane SADR-NABAVI

**Affiliations:** 1. Dept. of Medical Genetics, School of Medicine, Mashhad University of Medical Sciences, Mashhad, Iran; 2. Medical Genetic Research Center, School of Medicine, Mashhad University of Medical Sciences, Mashhad, Iran; 3. Dept. of Genetic, Academic Center for Education, Culture, and Research (ACECR), Mashhad, Iran; 4. Stem Cell and Regenerative Medicine Research Group, Academic Center for Education, Culture and Research (ACECR), Khorasan Razavi Branch, Mashhad, Iran

## Dear Editor-in-Chief

The world prevalence of intellectual disability (ID) has been reported to be 1%–3% of general population and most cases are seen in child/adolescent population (1, 2). The lifetime cost for each person with ID is approximately 1000000 dollars (1). In Iran, ID is mostly seen in adolescents and young people (13/1000) (2). Fragile X syndrome (FXS) is the most common cause of X-linked ID (3). According to Centers for Disease Control and Prevention (CDC) report, families became concerned about their children symptoms after first year of life and the diagnosis of FXS will be made a year after (4). Regarding the importance of FXS diagnosis and lack of clinical studies in our region, we decided to evaluate some features of children with ID referred with possible diagnosis of FXD to Genetic Center in Mashhad, Iran.

Individuals suggested for FXS diagnosis were assessed in a 4 yr period. Patients younger than 18 yr-old without family history of confirmed FXS or other psychiatric disorders were included. Informed consent was taken from participants and the study was approved by the local university.

**Fig. 1:**
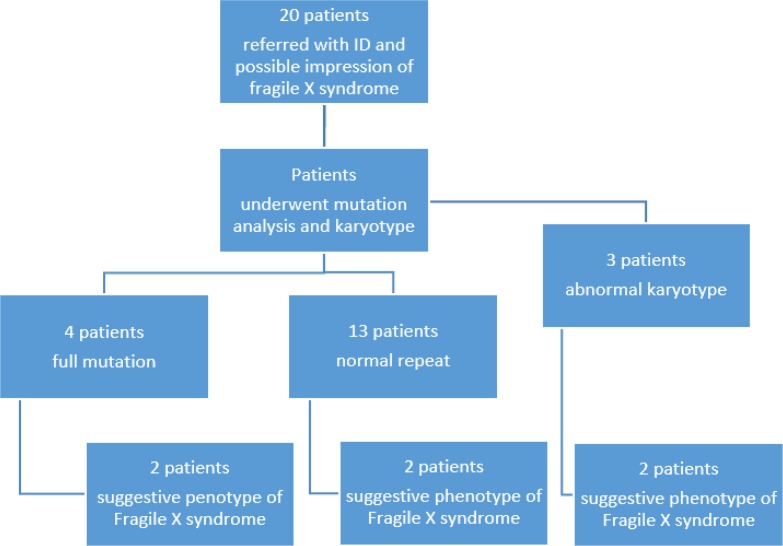
Distribution of suggestive phenotype according to genetic tests

The FXS testing was performed using Southern blot analysis with double digestion system. Twenty patients (2 female) with mean±SD age of 10.2±1.19 yr old enrolled and six patients had confirmed FXS and their mean±SD age was 11±6.13 yr old. Five patients had history of ID other than FXS in their family. Having relatives with ID was not associated with having full mutation (*P*=0.106). Only 30% of patients had suggestive phenotype of FXS but half of study population had full mutation. Among study population, 4 patients had array comparative genomic hybridization analysis. These 4 patients had normal results and normal triple repeats. Fifteen patients had karyotype which 3 of them were abnormal; showing features of fragile X chromosome (Fig. 1). Having suggestive phenotype is not associated with full mutation (*P*=0.33). The regression analysis showed that there was a significant positive relationship between CGG repeats and karyotype and diagnosis based on CGG cut-offs indicating that suggestive karyotype of fragile X chromosome was associated with 35.7% increase in number of CGG repeats and being categorized as positive diagnosis based on GCC repeat cut-offs was associated with 72% increase in number of CGG repeats.

Diagnosing the cause of ID is always a difficult issue for parents and physicians (3). Our study determined that 70% of patients with ID and suggestive diagnosis of FXS might have other causes for their ID except for FXS. A possible reason for this high rate of unexplained ID is the costs of genetic tests. Many families cannot offer further genetic tests for their children. Therefore, many suggested approaches for patients with ID will not be completed in our region.

FXS requires tight association between clinicians and medical geneticists for in time of diagnosis (5). According to CDC report, first sign of developmental delay is usually recognized by parents in first year and the clinical diagnosis is usually made in second year of life. The report stated that 24% of children with FXS had more than 10 visits before the genetic test was ordered (4). The mean referral age for genetic testing in our study was 11 yr old for confirmed cases which indicate delayed seeking for diagnosis. The exact reason of this late referral could not be established from this study; however, both clinicians and families could be responsible. Many of patients with FXS were visited by different specialist because of their ID or phenotypical features during their lifetime.

Delayed referral is also affected by syndrome’s features itself. Phenotype in males is variable and subtle patients will be difficultly detected in their prepubertal period. Developmental delay, mental retardation, and behavioral disturbances will help to establish diagnosis by 32 months of age (6). While all the children won’t have apparent features, every child presented with borderline ID, developmental delay or having autism without specific etiology should undergo testing for FXS (6). Nowadays, using computer software’s are becoming more prevalent in medical settings. There are some software developed for detection of genetic syndromes by scanning facial and phenotypical status of patients (7).

FXS is not widely studied in our region and according to our study delayed diagnosis is prominent. Reeducating medical professionals or using computer programs for recognition of genetic syndromes seems useful in reducing delayed diagnosis.
